# Cytochrome P450 and Glutathione S-Transferase Confer Metabolic Resistance to SYP-14288 and Multi-Drug Resistance in *Rhizoctonia solani*

**DOI:** 10.3389/fmicb.2022.806339

**Published:** 2022-03-21

**Authors:** Xingkai Cheng, Tan Dai, Zhihong Hu, Tongshan Cui, Weizhen Wang, Ping Han, Maolin Hu, Jianjun Hao, Pengfei Liu, Xili Liu

**Affiliations:** ^1^Department of Plant Pathology, China Agricultural University, Beijing, China; ^2^Institute of Quality Standard and Testing Technology, Beijing Academy of Agriculture and Forestry Sciences, Beijing, China; ^3^Shenzhen Agricultural Technology Promotion Center, Shenzhen, China; ^4^School of Food and Agriculture, University of Maine, Orono, ME, United States

**Keywords:** uncoupler, metabolic resistance, multi-drug resistance, *Rhizoctonia solani*, cytochrome P450s, glutathione-S-transferas

## Abstract

SYP-14288 is a fungicide as an uncoupler of oxidative phosphorylation, which is effective in controlling fungal pathogens like *Rhizoctonia solani.* To determine whether *R. solani* can develop SYP-14288 resistance and possibly multi-drug resistance (MDR), an SYP-14288-resistant mutant of *R. solani* X19-7 was generated from wild-type strain X19, and the mechanism of resistance was studied through metabolic and genetic assays. From metabolites of *R. solani* treated with SYP-14288, three compounds including M1, M2, and M3 were identified according to UPLC-MS/MS analysis, and M1 accumulated faster than M2 and M3 in X19-7. When X19-7 was treated by glutathione-S-transferase (GST) inhibitor diethyl maleate (DEM) and SYP-14288 together, or by DEM plus one of tested fungicides that have different modes of action, a synergistic activity of resistance occurred, implying that GSTs promoted metabolic resistance against SYP-14288 and therefore led to MDR. By comparing RNA sequences between X19-7 and X19, six cytochrome P450s (P450s) and two GST genes were selected as a target, which showed a higher expression in X19-7 than X19 both before and after the exposure to SYP-14288. Furthermore, heterologous expression of P450 and GST genes in yeast was conducted to confirm genes involved in metabolic resistance. In results, the P450 gene *AG1IA_05136* and GST gene *AG1IA_07383* were related to fungal resistance to multiple fungicides including SYP-14288, fluazinam, chlorothalonil, and difenoconazole. It was the first report that metabolic resistance of *R. solani* to uncouplers was associated with P450 and GST genes.

## Introduction

Plant diseases are an important constraint in crop production and food security (van Bruggen et al., 2016; Ristaino et al., 2021). For their management, synthetic fungicides are considered as an effective method (Ons et al., 2020; Raymaekers et al., 2020; Li et al., 2021). In the fungicide toolbox, uncouplers of oxidative phosphorylation have been widely used in the field due to their high-efficiency and broad-spectrum activities (Liang et al., 2015; Schepers et al., 2018). For example, fluazinam has shown a strong fungitoxicity against various phytopathogens by interrupting cellular energy production with an uncoupling activity on mitochondrial oxidative phosphorylation (Qu et al., 2018; Hou et al., 2019). As a potent uncoupler, fluazinam exhibited an excellent *in vitro* fungicidal activity on both mycelial growth and spore germination. It is registered for controlling diseases of root swelling of Chinese cabbage, late blight and early blight of potato, and anthracnose of pepper (Arjona-López et al., 2020; Li et al., 2020).

SYP-14288, or 2,4-dinitro-5-chloro-[1-(2,6-dichloro-4-nitroaniline)]-toluene (Shenyang Sinochem Agrochemicals R&D Co., Ltd., in China) shares a similar chemical structure with fluazinam and exhibits even a higher level of fungitoxicity than fluazinam against many plant pathogens (Wang et al., 2018; Cai et al., 2019). Similar to fluazinam, SYP-14288 has a mode of action (MoA) as an uncoupler that affects ATP biosynthesis by destroying the coupling of oxidative phosphorylation, which has been demonstrated in *Phytophthora capsici* (Wang et al., 2020) and *R. solani* (Liang et al., 2020).

Rapid adaption and resistance development in plant pathogens caused by intensive and overzealous applications of the same fungicides have quickly reduced fungicide efficiency (Takahashi et al., 2020; Tleuova et al., 2020). Although fungal resistance to uncouplers develops slowly, the risk of resistance is still a concern. An example is that fluazinam resistance has been reported in *Botrytis cinerea* (Tamura, 2000) and *Ustilago maydis* (Vitoratos, 2014). Moreover, a laboratory-generated mutant of *R. solani* resistant to SYP-14288 has cross-resistance with fungicides having not only the same but also different MoAs, indicating a multi-drug resistance (MDR) occurs (Cheng et al., 2020).

Modifications and over-expression of target genes and enhanced fungicide efflux are three known mechanisms of fungicide resistance in filamentous fungi (Gressel, 2020; Massi et al., 2021). However, it is uncertain if that of uncouplers falls in this category as there is no specific target protein or binding site corresponding to fungicides. Attempts have been made to explore the mechanism of fluazinam resistance. A positive cross-resistance between fluazinam and either procymidone or fludioxonil has been observed, and it is speculated that a similar resistance mechanism exists among these fungicides. However, no genetic mutations have been found in the nucleotide sequence of F-ATPase gene (Shao et al., 2015). Mao et al. (2018) have found that MAP/histidine kinases in osmotic signal transduction are involved in fluazinam resistance in *Sclerotinia sclerotiorum*, but neither mutations in *Shk1* of fluazinam-resistant mutants were found nor all of the expression level of three fluazinam-resistant mutants increased (Mao et al., 2018). Therefore, the specific resistance mechanism to uncouplers still remains unknown.

Another mechanism of fungicide resistance is over-expression of efflux transporters in plasma membranes, with the ATP-binding cassette (ABC) transporter or major facilitator superfamilies (MFS) commonly involved. They translocate invaded fungicides and efflux outside fungal cells using the energy either from ATP hydrolysis or from the proton-motive force, respectively (de Waard et al., 2006; Perlin et al., 2014). These transporters exhibit low substrate specificity, and their over-expression leads to simultaneous resistance to many structurally and functionally unrelated toxic compounds, which is a common MDR (Kretschmer et al., 2009; Omrane et al., 2015; Sang et al., 2015; Samaras et al., 2020; Khunweeraphong and Kuchler, 2021).

Cytochrome P450 monooxygenases (P450) and carboxylesterases (CarEs) belong to phase I metabolizing enzymes that catalyze xenobiotics and endobiotics mainly through hydroxylation/oxidation reactions (Basiliere and Kerrigan, 2020). P450s are heme-thiolate proteins found in all organisms and catalyze regio- and stereospecific conversions of a wide range of lipophilic compounds to more hydrophilic derivatives (Črešnar and Petrič, 2011). Collectively, the involvement of P450s has been proven in the metabolism of aliphatic, alicyclic, and aromatic molecules in reactions with *in situ* selectivity in hydroxylation, epoxidation, dealkylation, sulfoxydation, deamination, desulphuration, dehalogenation, and nitro reduction (Guengerich et al., 2016; Guengerich, 2021). Glutathione S-transferases (GSTs) and UDP-glucosyltransferases (UGTs) fall into phase II conjugating enzymes that add polar molecules onto compounds, producing water-soluble, non-toxic metabolites (Xu et al., 2005). GST-mediated detoxification can be derived either *via* direct metabolism or by the metabolism of secondary metabolites generated from phase I detoxification enzymes, as well as indirectly by providing protection against oxidative stress induced by xenobiotics exposure (Perperopoulou et al., 2017; Kumar and Trivedi, 2018). The over-expression of a P450 is involved in the resistance to neonicotinoid insecticides in whitefly *Bemisia tabaci* regulated by a transcription factor (Yang et al., 2020). A single P450 gene in a cross-pollinated weed species *Lolium rigidum* confirms that it could confer resistance to herbicides of at least five modes of action across seven herbicide chemistries (Han et al., 2021). However, research of metabolic resistance to fungicides in filamentous fungi has been rarely elucidated.

The goal of this study was to determine whether pathogenic fungi could develop resistance to uncouplers by enhancing the ability of detoxification and metabolism, which leads to MDR. Specific objectives were to (i) validate the role of phase I or II enzymes involved in SYP-14288 resistance and (ii) confirm the involvement of key detoxification genes in conferring SYP-14288 resistance.

## Materials and Methods

### Fungal Isolates and Chemicals

*Rhizoctonia solani* wild-type strain X19 was collected from rice plant, and SYP-14288-resistant mutant X19-7 with resistance factor being 56.7 was obtained through domestication of the parental strain X19 by exposing the culture to SYP-14288-amended media. X19-7 was not only resistant to SYP-14288 but also to fluazinam, fludioxonil, difenoconazole, chlorothalonil, and carbendazim (Cheng et al., 2020). In addition, sequence analysis showed that there were no mutations on the common target site including β*-tubulin*, *cytb*, *sdhB*, and histidine kinase gene of X19-7 compared with X19. Both cultures were preserved in the laboratory of Fungicide Pharmacology and Pathogen Resistance in China Agricultural University. Fungicides included SYP-14288 (active ingredient 97.0%), fluazinam (98.4%), chlorothalonil (98.5%), difenoconazole (98.4%), and azoxystrobin (98.0%). SYP-14288 was purified by silica gel column chromatography with elution by ethyl acetate and petroleum ether to a purity greater than 99% for its metabolic products analysis.

### Metabolism of SYP-14288 in *Rhizoctonia solani*

X19 and X19-7 mycelia were collected from cultures grown on potato dextrose agar (PDA), and approximately 0.1 g mycelia were cultured in 200-ml potato dextrose broth (PDB) for 48 h. After incubation, mycelia were harvested through a multifunction vacuum pump (Zhenjie Experimental Equipment Co., Ltd., Shanghai, China) at 0, 1, 3, 6, 12, and 24 h after addition of SYP-14288 at 0.1 μg/ml for X19-7 and 0.005 μg/ml for X19. The mycelia were freeze-dried in a freezer (Christ, Osterode, Germany) and stored at –80^°^C until use.

The treated mycelia were ground to powder in a ball mill (Restsch, Haan, Germany). Around 0.05 g powder was placed into a 2-ml centrifuge tube and suspended with 1.5 ml acetonitrile as extraction solution and 0.01 g primary secondary amine (PSA) to remove impurities such as organic acids, pigment, and sugars. The sample was vigorously shaken on an MX-F vortex mixer (Dragon Laboratory Instruments Ltd., Beijing, China) for 1 min before dissolution by an ultrasonicate (Kunshan Ultrasonic Instruments Co., Ltd., Kunshan, China) for 20 min. After centrifugation at 12,000 × *g* for 5 min, the supernatant was passed through a 0.22 μm membrane filter and collected in a centrifuge tube.

The metabolites were identified using a LC 1260/Q-TOF-MSMS 6520 system (Agilent Technologies, Santa Clara, CA, United States) with an Agilent electrospray ionization (ESI) with negative ion mode. Qualitative Analysis B.07.00 (Agilent Technologies, Santa Clara, CA, United States) was used to acquire mass spectrometric data. A method based on quick, easy, cheap, effective, rugged, and safe (QuEChERS) sample preparation coupled with UPLC-MS/MS was developed for detecting the content of SYP-14288 and its associated metabolites. An ACQUITY UPLC system (Waters, Milford, MA, United States) interfaced with a triple quadrupole mass spectrometer (TQS) that is equipped with an ESI source was employed for UPLC-MS/MS analyses (Wang et al., 2021). A flow rate of 0.3 ml/min at 40^°^C was maintained for chromatographic separation by using an ACQUITY UPLC BEH C18 column (2.1 mm × 100 mm, 1.8 μm; Waters, Wexford, Ireland). The mobile phase was 5 mM ammonium formate in methanol (A) and in ultrapure water (B), respectively. The metabolites were eluted on a linear solvent gradient program (10 min run time) using the following steps: linear gradient 65–95% solvent A (0–1 min), 95–95% A (1–7 min), 95–65% A (7.0–7.1 min), and 65–65% A (7.1–10 min). Detection was back to initial composition within 30 s and then equilibration for 2.5 min before the next injection (5 μl). The mass spectrometer was operated at the following settings: collision-induced desolvation temperature at 400^°^C; desolvation gas flow at 800 l/h; capillary voltage of 3.5 kV; and source temperature at 150^°^C. Optimized multiple reaction monitoring (MRM) transitions, retention times, collision energy (CE), and declustering potentials (DP) of the analytes were specified (Table 1). Mass spectrometric data were acquired and analyzed with corresponding software (Waters).

**TABLE 1:** Parameters used for high-performance liquid chromatography equipment in detecting SYP-14288 and associated metabolites.

Compound	Retention time (min)	Parent ion (m/z)	Daughter ion (m/z)	Collision energy (V)
M1	1.88	388.9562	200.0158	
			246.0100	22
			352.9868	
M2	1.91	399.9796	317.0028	20
			364.0023	18
M3	1.87	388.9637	292.9904	20
			322.9889	18
SYP-14288	1.98	418.9800	322.8300	
			342.7900	18
			382.7800	

*Dwell time was 20 s and cone voltage was 38 V.*

### Transcriptomic Assay

X19 and X19-7 either treated with SYP-14288 or non-treated were used for RNA-Seq analysis. An agar plug (5 mm in diameter) of culturen cut from the periphery of a 5-day-old colony was placed into 100 ml of PDB in a 250-ml flask and incubated at 25°C on a rotary shaker at 120 rpm for 2 days. The cultures of X19 and X19-7 were treated with SYP-14288 at 0.005 μg/ml and at 0.1 μg/ml, respectively, and incubated for additional 3 days. PDB free of SYP-14288 was used for control.

RNA was extracted using the Eastep Supper Total RNA Extraction Kit (Promega, Shanghai, China) according to the manufacturer’s procedure. Total RNA degradation and contamination were monitored on 1% agarose gels, and the RNA amount and purity of each sample was quantified using NanoPhotometer NP80 Touch (IMPLEN, Germany). RNA integrity was evaluated with a 2100 Bioanalyzer (Agilent Technologies, Santa Clara, CA, United States). The highest-quality RNA samples of three biological replicates were selected for the preparation of cDNA library. The transcriptome library construction and the Illumina sequencing were performed on an Illumina Novaseq™ 6000 (LC-Bio Technology Co., Ltd., Hangzhou, China) to yield 2 × 150 bp paired-end sequencing (PE150) following the vendor’s recommended protocol. Raw reads containing adaptor contamination were removed by Cutadapt software,^1^ and high-quality clean reads were used to map to the reference genome of *R. solani* AG1 IA available in RSIADB^2^ (Chen et al., 2016) and NCBI databases. The mapped reads of each sample were assembled using StringTie^3^ with default parameters. All transcriptomes of the samples were merged to reconstruct a comprehensive transcriptome, and after the final transcriptome was generated, StringTie and ballgown were used to estimate the expression levels of all transcripts and perform expression level for mRNAs by calculating fragments per kilobase million (FPKM). Differentially expressed genes (DEGs) among different RNA samples were selected based on fold change > 2 or fold change < 0.5 and *p*-value < 0.05 by R package, and the DEGs were analyzed by gene ontology (GO^4^ and Kyoto Encyclopedia of Genes and Genomes (KEGG)^5^ database enrichment analysis.

### Selection of Candidate Genes for Metabolic Resistance Through Gene Expression

To verify the reliability of RNA-seq data, DEGs of candidate resistance genes were selected for quantitative real-time polymerase chain reaction (qRT-PCR). Total RNA was extracted as described above. cDNA was synthesized using the 5X All-In-One MasterMix Kit (abm, Vancouver, BC, Canada), which was used as a template for qRT-PCR. The qRT-PCR was performed with various primers (Supplementary Table 1) on a qTOWER 2.2 system (Analytik Jena AG, Jena, Germany). Reactions were prepared using the SYBR Premix Dimer Eraser kit (Takara) according to the manufacturer’s instructions. Thermal cycler settings included denaturation at 95°C for 2 min, followed by 40 cycles of 95°C for 10 s and 60°C for 34 s. The samples were analyzed in a triplicate. The 2^–ΔΔCt^ method was employed to calculate the relative gene expression levels and data were analyzed with the Student’s *t*-test (*p* < 0.05) using SPSS (IBM, Armonk, NY, United States) (Livak and Schmittgen, 2001). β*-Actin* and *GAPDH* genes were used as reference to normalize quantification of the target gene expression.

### Synergy of Diethyl Maleate and Fungicides on Fungicide Resistance

To investigate the role of GST in SYP-14288 resistance as well as MDR mechanism, the GST enzyme inhibitor diethyl maleate (DEM) was selected for assessment of its interactions with SYP-14288 and other fungicides with different MoAs. DEM, SYP-14288, fluazinam, chlorothalonil, difenoconazole, and azoxystrobin were dissolved in dimethyl sulfoxide (DMSO) to make stock solutions (1 × 10^5^ μg/ml) and further diluted in a serialized manner to get several solutions of variable concentrations. For azoxystrobin, 100 μg/ml of salicylhydroxamic acid (SHAM) was added to suppress the alternative oxidase pathway. Effective concentration for 50% inhibition (EC_50_) of X19-7 to DEM and fungicides were first determined based on mycelial growth assay (Rebollar-Alviter et al., 2007). The fungicides and DEM were incorporated into the nutrient medium with a weight ratio (w:w) of (2–3000):1 for SYP-14288, (2–2000):1 for fluazinam, (2–200):1 for chlorothalonil, (2–3000):1 for difenoconazole, and (2–4000):1 for azoxystrobin, respectively. A bioassay was performed as described above and EC_50_ of mycelial growth were determined. Interactions between DEM and fungicides were evaluated by calculating the synergy ratio (SR) values using the following formula:

EC_50_(TH) = (a + b)/[a/EC_50_(A) + b/EC_50_(B)], where A and B represent fungicides with different MoAs and DEM, and a and b represent the ratio of these two components in the mixture. The level of SR is calculated as SR = EC_50_(TH)/EC_50_(OB), in which EC_50_(OB) is the observed EC_50_ value of the specific mixture (Wadley, 1945; Stergiopoulos and de Waard, 2002). Synergy was considered significant if iR ≥ 1.5, and antagonism if iR ≤ 0.5; additive interactions were considered to occur when 0.5 < iR < 1.5 (Gisi et al., 1985).

### Heterologous Expression of Cytochrome P450 and Glutathione-S-Transferase Genes in Yeast

To express the target genes in yeast, full-length cDNA sequences of P450 and GST genes were amplified using PCR. Primers for the amplification were designed with the Takara In-Fusion Cloning Primer Design Tool,^6^ which introduced *Eco*RI and *Xba*I sites at the 5’ and 3’ ends of the amplified product, respectively. Gene amplification was performed using EasyTaq^®^ DNA Polymerase (TransGen, Beijing, China) in a 50 μl mixture. Thermal cycler settings included an initial denaturation at 94°C for 5 min, followed by 35 cycles of denaturation at 94°C for 30 s, annealing at 58°C for 30 s, and extension at 72°C for 2 min, which was ended with an extension at 72°C for 5 min. PCR products were retrieved using the Gel Extraction Kit (CWBIO, Beijing, China) and the concentrations were measured with NanoPhotometer NP80 Touch. Meanwhile, plasmid pYES2/CT (Invitrogen, Carlsbad, CA, United States) was first digested with *Eco*RI and *Xba*I, gel-purified, and ligated with the above two products using ClonExpress^®^ Ultra One Step Cloning Kit (Vazyme, Nanjing, China) and then transformed into *Escherichia coli* DH5a cells following the manufacturer’s instructions. The culture was incubated on a Luria-Bertani solid medium containing 100 μg/ml ampicillin. A single colony in white color was picked for PCR and sequenced at the Tsingke Biotechnology Co. (Beijing, China). Heterologous expression in yeast was conducted based on the Yeastmaker Yeast Transformation System 2 which provides a high-efficiency polyethylene glycol (PEG)/LiAc-based method with minor modification (Takara, CA, United States).

*Saccharomyces cerevisiae* strain BY4741 (MATa his3Δ1 leu2Δ0 met15Δ0 ura3Δ0) was transferred into yeast peptone dextrose (YPD) (containing 2% glucose, 1% yeast extract, and 2% Bacto peptone) in a 50 ml tube and incubated at 30^°^C on a shaker at 200 rpm for 12 h. An aliquot of 5-μl culture was transferred into 5 ml of YPD in a 50-ml tube and continued incubating until the OD_600_ reached 0.6–0.8. The culture was collected after centrifugation at 3,040 × *g* for 2 min followed by washing with 10 ml 0.1 mol/l LiAc. The supernatant was discarded, and the cells were resuspended in 2.5 ml ddH_2_O, centrifuged at 3,040 × *g* for 2 min and collected, and later transferred to 1.5 ml tubes, resuspended with 50 μl ddH_2_O and centrifuged at 9,600 × *g* for 15 s to obtain the cells.

Solid synthetic dextrose (SD) minimal medium containing 26.7 g/l minimal SD base (no amino acids), 0.77 g/l drop-out mix containing amino acids minus uracil, 20 g/l glucose, and agar 20 g/l was used for the growth of yeast transformants. A transformation mixture composed of 240 μl of PEG, 36 μl of LiAc, 50 μl of ssDNA, 1 μg plasmid DNA, and ddH_2_O were added into the tube, mixed thoroughly, and placed on ice. The tubes were incubated in a 42^°^C bath for 40 min, then centrifuged at 1,500 × *g* for 2 min to remove the supernatant and resuspended in 150 μl 0.9% (w/v) NaCl solution. Transformed cells were spread onto an SD plate and incubated at 30 C until colonies appear. The colony was selected for further verification.

*Saccharomyces cerevisiae* expression vector pYES2/CT (Invitrogen, Carlsbad, CA, United States) was used for gene transformation. pYES2/CT carrying P450 genes *AG1IA_05136* (Accession: 443921058), *AG1IA_07929* (Accession: 443917283), *AG1IA_01023* (Accession: 443926231), *AG1IA_06336* (Accession: 443919487), *AG1IA_07129* (Accession: 443918339), and GST gene *AG1IA_07383* (Accession: 443917998) were cultured at 30^°^C for 3 days in liquid SD modified by replacing uracil with 2% galactose. Yeast transformants with the empty vector pYES2/CT were used as a control. Cell suspensions were diluted to an OD_600_ of 0.5 in liquid SD, which was measured using the NanoPhotometer NP80 Touch, and 5 μl of each yeast transformant was plated as spots on SD agar medium lacking uracil, containing 2% galactose and amended with different concentrations of fungicides. The sensitivity of yeast transformants to fungicides was qualitatively assessed after incubation at 30^°^C for 3 days. The fungicide concentrations (μg/ml) used in the study were as follows: SYP-14288 (0, 0.1, 1, and 10), fluazinam (0, 0.1, 1, and 10), chlorothalonil (0, 0.1, 1, and 5), and difenoconazole (0, 0.1, 1, and 10). Two biological replicates and four technical replicates per biological replicate were assigned for each transformant and treatment.

## Results

### Metabolism of SYP-14288 in *Rhizoctonia solani*

**FIGURE 1:**
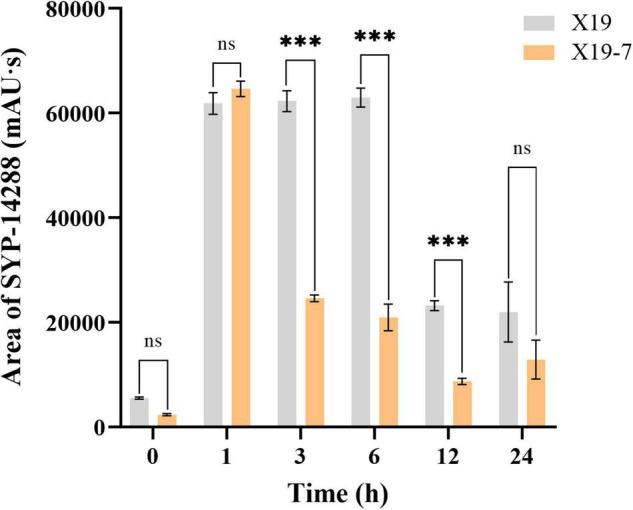
Content of SYP-14288 in mycelia of *Rhizoctonia solani* wild-type strain X19 and SYP-14288-resistant mutant X19-7, which were treated with 0.005 and 0.1 μg/ml SYP-14288, respectively. SYP-14288 was quantified at 0, 1, 3, 6, 12, and 24 h after treatment in high-performance liquid chromatography equipment. Values are mean ± SD of at least three independent experiments, and data were analyzed with ANOVA followed by Fisher’s least significant difference (LSD) test (^***^p < 0.01; ns, non-significant).

SYP-14288 content in hyphae was significantly reduced in the SYP-14288-resistant mutant X19-7 compared with its parental strain X19 (wild-type) under SYP-14288 treatment over time (Figure 1). The content did not show a significant difference between the two strains at the beginning of treatment. Both reached the maximum within 1 h, which implied a similar absorption rate between mutant and wild-type strains. However, SYP-14288 was sharply reduced more in X19-7 than in X19 after 3, 6, and 12 h of treatment. SYP-14288 content in X19-7 and X19 tended to be stable with the extension of treating time, and the difference between them was less significant after 24 h. Thus, the reduction of SYP-14288 in X19-7 might be caused by efflux or detoxification metabolism.

**FIGURE 2:**
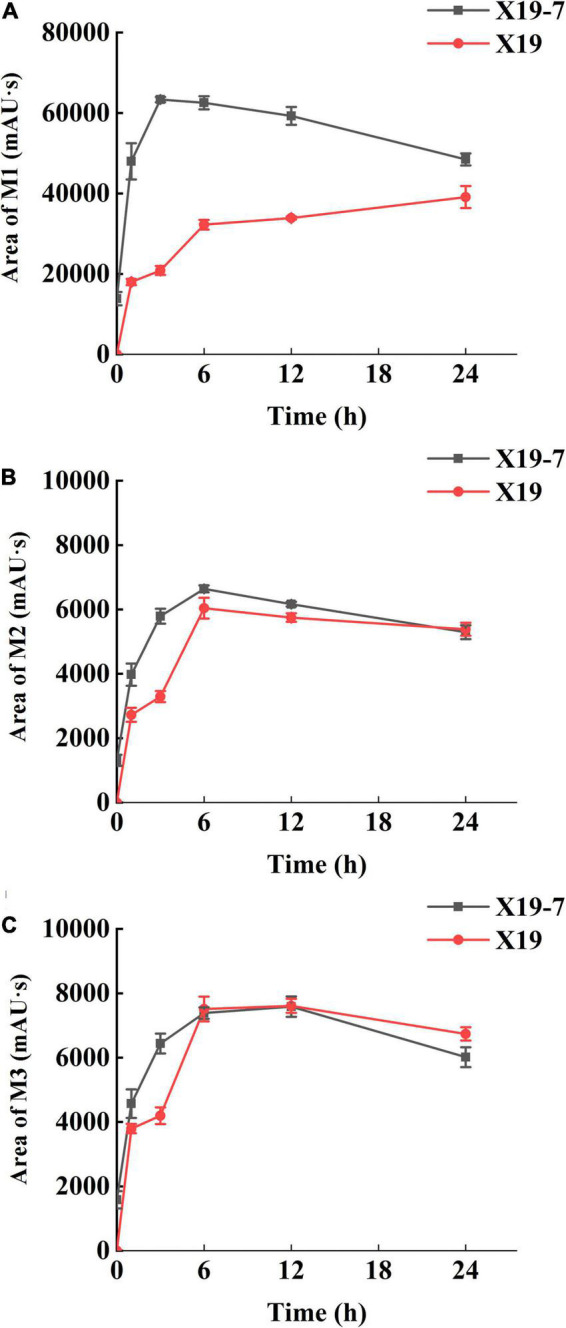
Quantification of SYP-14288 metabolites M1 **(A)**, M2 **(B)**, and M3 **(C)** in the mycelia of *Rhizoctonia solani* X19 and SYP-14288-resistant mutant X19-7. SYP-14288 was quantified at 0, 1, 3, 6, 12, and 24 h after treatment in ultra-performance liquid chromatography-tandem mass spectrometry. Values are mean ± SD.

The metabolome of SYP-14288 both in the resistant mutant X19-7 and parental isolate X19 was analyzed by UPLC-MS/MS, and three metabolites including M1, M2, and M3 were identified according to the optimized ion source parameters (Table 1). The molecule ion *m/z* of M1 and M3 was 388.9562 [M-H^+^] and 388.9637 [M-H^+^] respectively, which showed a loss of *m/z* 30.0238 and 30.0163 compared to SYP-14288 (*m/z* 418.9800 [M-H^+^]). This indicated a reduction from -NO_2_ to -NH_2_. Molecule ion *m/z* of M2 was 399.9796 [M-H^+^], with reduced *m/z* 19.00 compared with SYP-14288, which implied a probable substitution of -Cl with -NH_2_. In addition, these three metabolites were analyzed quantitatively at different treating time. The content of the three metabolites M1, M2, and M3 in both X19-7 and X19 increased at the beginning of treatment but reduced as the time of treatment extended, except for M1 in X19, which showed a constant increase (Figure 2). M1 content in X19-7 was much higher than that in X19 and exhibited the most significant difference of 3.04 folds (Figure 2A). Content of M2 showed a similar trend with M1. However, content difference between X19-7 and X19 was not significant compared to M1 and had no difference at the end of treatment (Figure 2B). There was no significant difference in the accumulation of metabolite M3 between X19-7 and X19 at most time points and even lower than X19 during some treatment time periods (Figure 2C). Results showed that detoxification metabolism existed both in parental isolate and resistant mutant which caused reaction reduction of -NO_2_ and substitution of -Cl in SYP-14288.

### Transcriptomic Assay

RNA was sequenced to define the transcriptome of *R. solani* involved in the SYP-14288 resistance phenotype. A total of 191,774,723 raw sequence reads were generated from the assembled RNA-Seq data of 12 RNA libraries, including untreated X19 and X19-7, and SYP-14288-treated X19 (X19T) and X19-7 (X19-7T). For each RNA-seq library, over 40 million reads were produced and nearly 80% were mapped back in pairs for both biological replicates of treated and untreated samples of X19 and X19-7. About 187,884,585 clean reads resulting from quality control procedures were used for *de novo* assembly (Supplementary Table 2). Results demonstrated that the higher sequencing quality of the mapped reads was correlated with better assembly integrity and met the demand for subsequent quantitative expression and annotation analysis. UniGenes were compared with GO and KEGG analysis to obtain annotation information, and of the assembled contigs, six up-regulated genes were significantly enriched in the “drug metabolizing cytochrome CytP450,” showing high over-expression in both X19-7 and X19-7T when compared with X19. Two genes were identified belonging to “drug metabolizing cytochrome GST” (Table 2).

**TABLE 2:** Transcriptome contigs encoding cytochrome P450 and glutathione S-transferase genes in wild-type strain X19 and SYP-14288-resistant mutant X19-7 of *Rhizoctonia solani.*

Gene ID	PFAM^a^ accession	Function annotation	*P* ^b^	Fold change between X19-7 and X19^c^
gene-AG1IA_05136	PF00067.16	Cytochrome P450	2.87 × 10^–5^	4.49
gene-AG1IA_01023	PF00067.16	Cytochrome P450	7.44 × 10^–4^	3.15
gene-AG1IA_05092	PF00067.16	Cytochrome P450	7.79 × 10^–12^	37.84
gene-AG1IA_07129	PF00067.16	Cytochrome P450	1.14 × 10^–3^	5.23
gene-AG1IA_07929	PF00067.16	Cytochrome P450	6.34 × 10^–6^	6.26
gene-AG1IA_06336	PF00067.16	Cytochrome P450	1.70 × 10^–8^	45.75
gene-AG1IA_07383	PF00043.19	Glutathione S-transferase	2.29 × 10^–5^	10.25
gene-AG1IA_00711	PF02798.14	Glutathione S-transferase	2.17 × 10^–7^	28.95

*^a^PFAM represents the protein families database.*

*^b^P is expressed by the Benjamini–Hochberg procedure for controlling the false discovery rate.*

*^c^Fold change = 2^–Δ^ ^ΔCt^, where ΔCt = Ct (P450 or GST genes) – Ct (reference genes), and ΔΔCt = ΔCt (X19-7) – ΔCt (X19).*

### Selection of Metabolic-Resistance Genes

**FIGURE 3:**
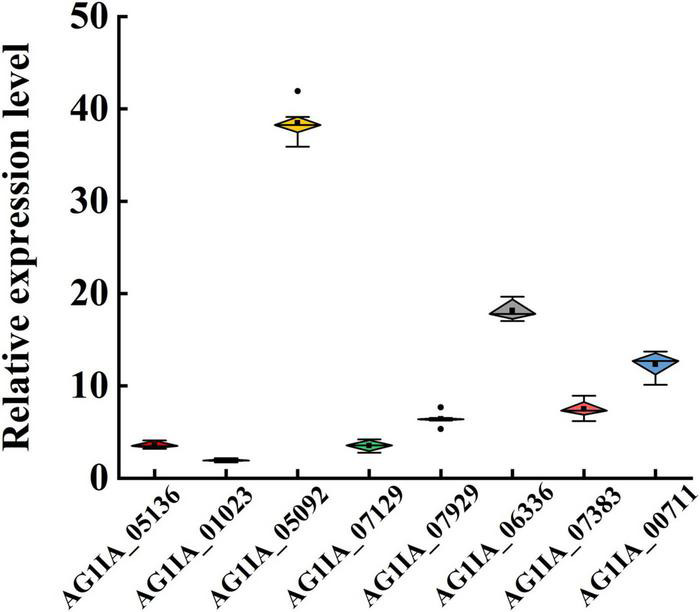
Relative expression of cytochrome P450 genes *AG1IA_05136*, *AG1IA_01023*, *AG1IA_07129*, *AG1IA_07929*, and *AG1IA_06336* and glutathione transferases gene *AG1IA_07383* in SYP-14288-resistant mutant X19-7 and its parental isolate X19 of *Rhizoctonia solani*. Values were mean ± SD of at least three independent experiments, and data were analyzed with ANOVA followed by Fisher’s least significant difference (LSD) test (significance level α = 0.01).

To validate candidate genes involved in metabolic resistance to SYP-14288, expression of genes upregulated in treated strains X19-7 and X19 with similar functional annotations (i.e., encoding P450s and GSTs) were selected and quantified by qRT-PCR. Among the P450s, mRNA levels of *AG1IA_05136*, *AG1IA_01023*, *AG1IA_05092*, *AG1IA_07129*, *AG1IA_07929*, and *AG1IA_06336* were consistent with the RNA sequence data and a significantly higher up-regulation with 3.58, 38.27, 3.51, 6.39, and 18.05-fold in the X19-7 compared to X19, respectively. Like P450s, mRNA levels of two GST genes *AG1IA_07383* and *AG1IA_00711* were 7.44 and 12.26-fold in the X19-7, respectively (Figure 3).

### Synergy of Diethyl Maleate and Fungicides on Fungicide Resistance

**FIGURE 4:**
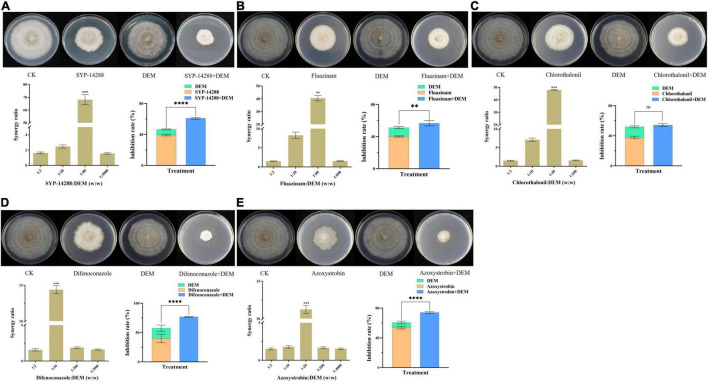
Growth inhibition of *Rhizoctonia solani* X19-7 by fungicides and their combination with diethyl maleate (DEM). X19-7 was cultured on potato dextrose agar (PDA). Treatments included SYP-14288 **(A)**, fluazinam **(B)**, chlorothalonil **(C)**, difenoconazole **(D)**, and azoxystrobin **(E)** amended in PDA. Fungicide-free PDA was used for control. Data were presented as mean ± SD, and the same strain followed by different letters was significantly different according to Fisher’s least significant difference (LSD) test (significance level α = 0.05). Significant differences from mean values of DEM treatment are indicated by asterisks: ^**^when *p* < 0.01, ***when *p* < 0.005, ^****^when *p* < 0.001, and ns when there was no significance.

To determine the role of GSTs in metabolic resistance, DEM and fungicides were investigated by co-application. Mycelial growth of X19-7 was less affected by DEM but significantly inhibited when treated with the combination of DEM and SYP-14288, fluazinam, chlorothalonil, difenoconazole, or azoxystrobin, which was much higher than the fungicide applied alone in most cases (Figure 4). SR values were 1.63, 2.51, 68.16, and 1.56 under different weight ratios of 1:2, 1:20, 1:80, and 1:3000 between SYP-14288 and DEM, respectively, showing a synergistic activity. When the ratio of SYP-14288 and DEM was 1:80, SR value reached a maximum of 68.16. Furthermore, inhibition rate for SYP-14288 against X19-7 was 61.0% in the presence of DEM, which was much higher than SYP-14288 treatment alone with inhibition rate of 39.4% (Figure 4A). Similarly, synergistic activity was observed between DEM and fluazinam, which has the same MoA with SYP-14288, and the SR value was highest at 40.50 in a 1:80 weight ratio. Inhibition rate for fluazinam was lower when combined with DEM than SYP-14288 with being 56.7% and exhibited a 16.5% enhancement than fluazinam treatment alone (Figure 4B). In addition, synergistic activity was also observed between fungicides with different MoAs and DEM. SR values reached maximum of 47.86 for chlorothalonil, 14.40 for difenoconazole, and 11.23 for azoxystrobin in different weight ratios of 1:40, 1:20, and 1:20, respectively (Figures 4C–E). All above results indicated that GSTs were involved in SYP-14288 resistance and may play a vital role in MDR.

### Heterologous Expression of P450 and Glutathione-S-Transferase Genes in *Saccharomyces cerevisiae*

**FIGURE 5:**
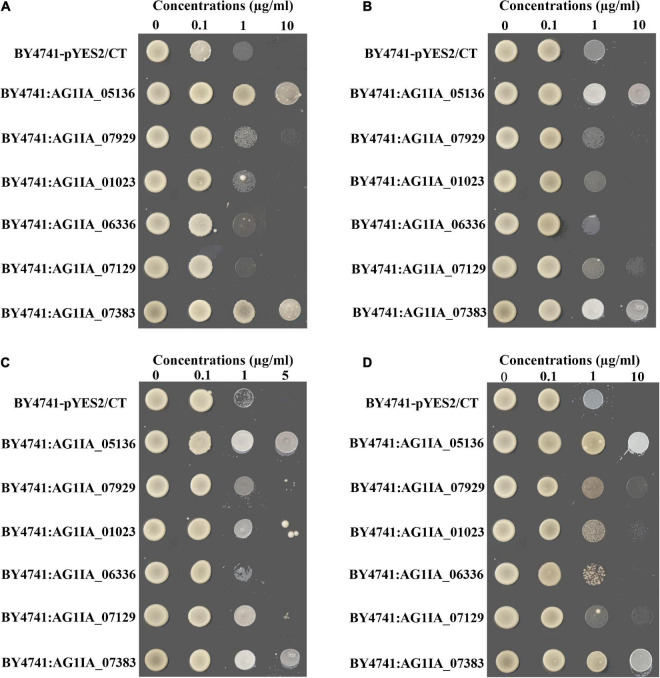
Heterologous expression of the cytochrome P450 genes *AG1IA_05136*, *AG1IA_01023*, *AG1IA_07129*, *AG1IA_07929*, and *AG1IA_06336* and glutathione transferase gene *AG1IA_07383* in a drug-hypersensitive *Saccharomyces cerevisiae* strain BY4741. The sensitivity of the transformants were tested on solid synthetic dextrose minimal agar medium lacking uracil, containing 2% galactose and amended with different concentrations of SYP-14288 **(A)**, fluazinam **(B)**, chlorothalonil **(C)**, and difenoconazole **(D)**. *Saccharomyces cerevisiae* strain BY4741 was transformed with expression vector pYES2/CT (BY4741-pYZS2/CT), with P450 genes *AG1IA_05136* (BY4741:AG1IA_05136), *AG1IA_07929* (BY4741:AG1IA_07929), *AG1IA_01023* (BY4741:AG1IA_01023), *AG1IA_06336* (BY4741:AG1IA_06336), and *AG1IA_07129* (BY4741:AG1IA_07129) and with glutathione S-transferase gene *AG1IA_07383* (BY4741: AG1IA_07383).

The hypersensitive yeast *S. cerevisiae* strain BY4741 was transformed with full-length of P450 genes *AG1IA_05136*, *AG1IA_07929*, *AG1IA_01023*, *AG1IA_06336*, and *AG1IA_07129* and GST gene *AG1IA_07383*. Fungicide sensitivity was different between the two gene transformants (BY4741:AG1IA*_*05136 and BY4741:AG1IA_07383) and the control transformant (BY4741-pYES2/CT) when treated with SYP-14288, fluazinam, chlorothalonil, or difenoconazole (Figure 5). Both transformants and empty vector grew under low concentrations of SYP-14288. However, the growth of empty vector and other gene transformants was suppressed as the concentration increased while the transformants BY4741:AG1IA*_*05136 and BY4741:AG1IA_07383 were able to grow normally even at high concentration (Figure 5A). A similar trend occurred in treatments with fluazinam, chlorothalonil, and difenoconazole. Transformants BY4741:AG1IA*_*05136 and BY4741:AG1IA_07383 were able to constantly grow in the plates amended with different concentrations of fungicides, while other transformants stopped growing under high concentration (Figures 5B–D). Although colony of transformants BY4741:AG1IA_07929, BY4741:AG1IA_07129, and BY4741:AG1IA_01023 could be observed under high concentrations of fluazinam, chlorothalonil, and difenoconazole, growth of these transformants was significantly inhibited compared with BY4741:AG1IA_05136 and BY4741:AG1IA_07383. Therefore, the over-expression of P450 gene *AG1IA_05136* and GST gene *AG1IA_07383* contributed to resistance in the *R. solani* by phase I and II detoxification pathways, but other genes might not be involved in the resistance.

## Discussion

We have demonstrated that SYP-14288 treatment resulted in specific metabolism of *R. solani* that was promoted by over-expression of P450 genes, expressing fungicide resistance including MDR. P450s can catalyze the conversion of hydrophobic intermediates of primary and secondary metabolic pathways through hydroxylation, dehalogenation, dealkylation, and nitro reduction and therefore neutralize the highly reactive nucleophile sites of the chemical and/or increasing its water-solubility facilitating its excretion from the cell (Hannemann et al., 2007; Lu et al., 2021). Reduction of nitro compounds by P450s have been verified and previous study demonstrated that P450 could act as a reductase, catalyzing the reduction of the aromatic nitro group to the aniline (Guengerich, 2001; Pochapsky et al., 2018). There were three identified SYP-14288 metabolites, M1, M2, and M3, and based on catalytic properties of P450s and changes in the molecule ion *m/z*, a reduction from -NO_2_ to -NH_2_ of M1 was inferred to be existent in the SYP-14288 metabolism. Moreover, SYP-14288-resistant mutant X19-7 significantly enhanced SYP-14288 metabolism on M1, and M1 accumulation in X19-7 might account for the decrease of the sensitivity to SYP-14288, whereas M2 and M3 were less important in the resistance. Over-expression of the P450 gene *AG1IA_05136* significantly decreased the sensitivity to not only SYP-14288 but also fluazinam, chlorothalonil, and difenoconazole. Because these fungicides have different MoAs, the results indicated that MDR occurred. Therefore, over-expression of the P450 gene *AG1IA_05136* was associated with SYP-14288 metabolic resistance, and led to MDR. Other P450 genes showing a weak association may be due to different substrate specificity. Meanwhile, some genes in X19-7 possibly have mutations which could change catalytic activities of related enzymes like P450s, therefore leading to SYP-14288 resistance even though transcriptional levels were the same as in the wild-type strain.

Metabolic resistance is characterized by enhanced detoxification of xenobiotics and mediated by P450s. It is considered as a common type of metabolism-based resistance to insecticides and herbicides (Ffrench-Constant, 2013; Richter et al., 2016). A few pathogenic fungi have been confirmed to produce detoxification enzymes responsible for detoxification or resistance. In *Botrytis pseudocinerea*, a P450 gene *CYP684* is responsible for its resistance to fenhexamid due to the over-expression caused by a 25-bp deletion in the promoter or a 10-bp insertion in the 3’UTR of the gene (Azeddine et al., 2014). Over-expression of phase I cytochrome P450s and phase III ABCs was associated with the MDR of *S. homoeocarpa via* the xenobiotic detoxification pathway regulated by a gain-of-function mutation of the transcription factor ShXDR1 (Sang et al., 2018). P450-mediated metabolic pathway relates to the resistance of *Magnaporthe oryzae* to novel pyrimidine amines (Zhang et al., 2020). Our study confirmed the role of the P450 gene *AG1IA_05136* in SYP-14288 resistance was attributed to converting SYP-14288 to the corresponding metabolites, increasing its water solubility, and promoting excretion from *R. solani* cells.

Metabolic resistance can also be confirmed indirectly by using enzyme inhibitors. Certain P450 or GST inhibitors can effectively reverse metabolic resistance both in weeds and insects (Feyereisen, 2015; Pavlidi et al., 2018). The over-expression of the cytochrome P450 gene *CYP6CM1* determines imidacloprid resistance of *Bemisia tabaci*, and piperonyl butoxide (PBO) is a diagnostic attribute of metabolic resistance through inhibition of cytochrome P450 enzymes (Panini et al., 2017). The GST inhibitor pharmacophore 4-chloro-7-nitro-benzoxadiazole is active toward *AmGSTF1* and used to restore herbicide efficacy in multiple-herbicide resistance in black-grass (Cummins et al., 2013). In this study, the GST inhibitor DEM exhibited a synergistic activity with SYP-14288 and other fungicides with different MoAs including fluazinam, chlorothalonil, difenoconazole, and azoxystrobin in resistant mutant X19-7. However, such synergism was not observed in the wild-type strain X19. Thus, GSTs can be used as a tool to confirm the potential resistance of *R. solani* to SYP-14288 and other fungicides and may be used as an effective measure to reverse MDR. GSTs are best known for catalyzing the conjugation of reduced glutathione (GS^–^) to substrates containing an electrophilic center, hereby increasing the substrate’s water solubility and, thus, facilitating its excretion from the cell (Oakley, 2011). They may participate in chemical pesticide detoxification either by direct metabolism by dehydrochlorination of organochlorines, O-dealkylation/O-dearylation, and glutathione conjugation of pesticides or by passive binding *via* sequestration (Gonzalez et al., 2018; Georgakis et al., 2020; Kouamo et al., 2021). Over-expression of the GST gene *AG1IA_07383* was observed in resistant mutant 19-7 of *R. solani*, and heterologous expression of the gene confirmed its contribution in *R. solani* resistance to SYP-14288. The role of *AG1IA_07383* in SYP-14288 resistance may involve in catalyzing the conjugation of GS^–^ to the metabolites derived from the P450 detoxification enzyme. The conjugates increased water solubility, reduced phytotoxicity, and made it easier to excrete out of cells. However, there is no sufficient evidence of GS^–^ binding to SYP-14288; therefore, how GSTs increase metabolic resistance in *R. solani* needs further investigation.

Several ABC and MFS transporters were also identified as they were constitutively over-expressed in the SYP-14288-resistant mutant either before or after the exposure to SYP-14288. This makes sense because ABC and MFS transporters are commonly associated with MDR in various types of pathogens (Borst, 2020; Drew et al., 2021). MDR caused by increased efflux activity has been described in phytopathogenic fungi. Kretschmer et al. (2009) have demonstrated that MDR phenotypes of *B. cinerea* are correlated with increased drug efflux activity and over-expression of ABC and MFS efflux transporters. Expression of the MgMFS1 gene was constitutively high in MDR *Z. tritici* field isolates, and a 519 bp insertion in the MgMFS1 promoter was detected in MDR field strains rather than sensitive strains, suggesting it contributed to the observed MDR phenotype (Omrane et al., 2015). Over-expression of efflux transporters genes implied that they were involved in SYP-14288-related metabolic resistance; however, no synergistic activities were observed between fungicide and more than 10 commonly efflux inhibitors. In addition, heterologous expression of these genes in *S. cerevisiae* showed no difference in sensitivity to SYP-14288, fluazinam, chlorothalonil, or difenoconazole between ABC and MFS genes transformants and empty vector, which demonstrated that efflux genes were not directly involved in fungicide resistance (data not shown). Nevertheless, the role of ABC and MFS should be considered in resistance, which may participate in this progress by enhancing the efficiency and ease in pumping fungicides out as SYP-14288 is degraded.

Multi-drug resistance induced by a single fungicide SYP-14288 extended our knowledge of fungicide resistance. It greatly challenged the management of fungicide resistance in fields as it can cause deficiency of multiple fungicides simultaneously. This phenomenon may overwrite our common sense that a fungicide resistance can be prevented by using fungicides with different MOAs. To effectively manage MDR, using corresponding metabolic enzyme inhibitors may be a potential strategy.

## Conclusion

In conclusion, it was the first report that metabolic resistance of *R. solani* to uncouplers was associated with P450 and GST genes. The over-expression of the P450 gene *AG1IA_05136* and GST gene *AG1IA_07383* in *R. solani* determined the detoxification of or resistance to SYP-14288. The specific regulation pathway between different detoxification phases needs further exploration. This suggests that metabolic resistance can be a significant threat to pesticide efficacy as it can automatically confer resistance to existing, new, or yet-to-be-discovered chemical pesticides. In practice, it is possible to use certain P450 or GST inhibitors to reverse metabolic resistance.

## Data Availability Statement

The data presented in the study are deposited in the SRA repository, accession number PRJNA778030.

## Author Contributions

PL, XL, and XC designed the experiments. XC performed the experiments and wrote the manuscript. TD and ZH participated in the experiment of metabolism of SYP-14288 in *Rhizoctonia solani*. TC and WW provided help for heterologous expression of P450 and GST genes in yeast. JH help revise the manuscript. All authors reviewed the manuscript.

## Conflict of Interest

The authors declare that the research was conducted in the absence of any commercial or financial relationships that could be construed as a potential conflict of interest.

## Publisher’s Note

All claims expressed in this article are solely those of the authors and do not necessarily represent those of their affiliated organizations, or those of the publisher, the editors and the reviewers. Any product that may be evaluated in this article, or claim that may be made by its manufacturer, is not guaranteed or endorsed by the publisher.

## Footnotes

1
https://cutadapt.readthedocs.io/en/stable/

2
http://genedenovoweb.ticp.net:81/rsia/

3
http://ccb.jhu.edu/software/stringtie/

4
http://www.geneontology.org/

5
http://www.kegg.jp/

6
https://www.takarabio.com/learning-centers/cloning/primer-design-and-other-tools

## References

[B1] Arjona-López J. M. Capote N. Melero-Vara J. M. López-Herrera C. J. (2020). Control of avocado white root rot by chemical treatments with fluazinam in avocado orchards. *Crop Prot.* 131:105100. 10.1016/j.cropro.2020.105100

[B2] Azeddine S. Billard A. Bach J. Lanen C. Walker A. S. Debieu D. (2014). “*Botrytis pseudocinerea* is resistant to the fungicide fenhexamid due to detoxification by the cytochrome P450 monooxygenase CYP684,” in *Proceedings of the 10es Rencontres de Phytopathologie-Mycologie de la Société Française de Phytopathologie (SFP)*, (Aussois: FAO), 83.

[B3] Basiliere S. Kerrigan S. (2020). CYP450-mediated metabolism of mitragynine and investigation of metabolites in human urine. *J. Anal. Toxicol.* 44 301–313. 10.1093/jat/bkz108 32008041

[B4] Borst P. (2020). Looking back at multidrug resistance (MDR) research and ten mistakes to be avoided when writing about ABC transporters in MDR. *FEBS Lett.* 594 4001–4011. 10.1002/1873-3468.13972 33111311

[B5] Cai M. Wang Z. Ni X. Hou Y. Peng Q. Gao X. (2019). Insights from the proteome profile of *Phytophthora capsici* in response to the novel fungicide SYP-14288. *PeerJ* 7:e7626. 10.7717/peerj.7626 31523524 PMC6716503

[B6] Chen L. Ai P. Zhang J. Deng Q. Wang S. Li S. (2016). RSIADB, a collective resource for genome and transcriptome analyses in *Rhizoctonia solani* AG1 IA. *Database* 2016:baw031. 10.1093/database/baw031 27022158 PMC4809263

[B7] Cheng X. Man X. Wang Z. Liang L. Zhang F. Wang Z. (2020). Fungicide SYP-14288 inducing multi-drug resistance in *Rhizoctonia solani*. *Plant Dis.* 104 2563–2570. 10.1094/PDIS-01-20-0048-RE 32762501

[B8] Črešnar B. Petrič Š (2011). Cytochrome P450 enzymes in the fungal kingdom. *BBA Proteins Proteom.* 1814 29–35. 10.1016/j.bbapap.2010.06.020 20619366

[B9] Cummins I. Wortley D. J. Sabbadin F. He Z. Coxon C. R. Straker H. E. (2013). Key role for a glutathione transferase in multiple-herbicide resistance in grass weeds. *Proc. Natl. Acad. Sci. U.S.A.* 110 5812–5817. 10.1073/pnas.1221179110 23530204 PMC3625300

[B10] de Waard M. A. Andrade A. C. Hayashi K. Schoonbeek H. J. Stergiopoulos I. Zwiers L. H. (2006). Impact of fungal drug transporters on fungicide sensitivity, multidrug resistance and virulence. *Pest Manag. Sci.* 62 195–207. 10.1002/ps.1150 16475240

[B11] Drew D. North R. A. Nagarathinam K. Tanabe M. (2021). Structures and general transport mechanisms by the major facilitator superfamily (MFS). *Chem. Rev.* 121 5289–5335. 10.1021/acs.chemrev.0c00983 33886296 PMC8154325

[B12] Feyereisen R. (2015). Insect P450 inhibitors and insecticides: challenges and opportunities. *Pest Manag. Sci.* 71 793–800. 10.1002/ps.3895 25404103

[B13] Ffrench-Constant R. H. (2013). The molecular genetics of insecticide resistance. *Genetics* 194 807–815. 10.1534/genetics.112.141895 23908373 PMC3730913

[B14] Georgakis N. Poudel N. Papageorgiou A. C. Labrou N. E. (2020). Comparative structural and functional analysis of phi class glutathione transferases involved in multiple-herbicide resistance of grass weeds and crops. *Plant Physiol. Bioch.* 149 266–276. 10.1016/j.plaphy.2020.02.012 32088578

[B15] Gisi U. Binder H. Rimbach E. (1985). Synergistic interactions of fungicides with different modes of action. *Trans. Br. Mycol. Soc.* 85 299–306. 10.1016/S0007-1536(85)80192-3

[B16] Gonzalez D. Fraichard S. Grassein P. Delarue P. Senet P. Nicolaï A. (2018). Characterization of a Drosophila glutathione transferase involved in isothiocyanate detoxification. *Insect Biochem. Mol. Biol.* 95 33–43. 10.1016/j.ibmb.2018.03.004 29578047

[B17] Gressel J. (2020). Perspective: present pesticide discovery paradigms promote the evolution of resistance-learn from nature and prioritize multi−target site inhibitor design. *Pest Manag. Sci.* 76 421–425. 10.1002/ps.5649 31613036

[B18] Guengerich F. P. (2001). Common and uncommon cytochrome P450 reactions related to metabolism and chemical toxicity. *Chem. Res. Toxicol.* 14 611–650. 10.1021/tx0002583 11409933

[B19] Guengerich F. P. (2021). A history of the roles of cytochrome P450 enzymes in the toxicity of drugs. *Toxicol. Res.* 37 1–23. 10.1007/s43188-020-00056-z 32837681 PMC7431904

[B20] Guengerich F. P. Waterman M. R. Egli M. (2016). Recent structural insights into cytochrome P450 function. *Trends Pharmacol. Sci.* 37 625–640. 10.1016/j.tips.2016.05.006 27267697 PMC4961565

[B21] Han H. Yu Q. Beffa R. González S. Maiwald F. Wang J. (2021). Cytochrome P450 *CYP81A10v7* in *Lolium rigidum* confers metabolic resistance to herbicides across at least five modes of action. *Plant J.* 105 79–92. 10.1111/tpj.15040 33098711

[B22] Hannemann F. Bichet A. Ewen K. M. Bernhardt R. (2007). Cytochrome P450 systems-biological variations of electron transport chains. *Biochim. Biophys. Acta* 1770 330–344. 10.1016/j.bbagen.2006.07.017 16978787

[B23] Hou Y. P. Mao X. W. Wu L. Y. Wang J. X. Mi B. Zhou M. G. (2019). Impact of fluazinam on morphological and physiological characteristics of *Sclerotinia sclerotiorum*. *Pestic. Biochem. Phys.* 155 81–89. 10.1016/j.pestbp.2019.01.009 30857631

[B24] Khunweeraphong N. Kuchler K. (2021). Multidrug resistance in mammals and fungi-from MDR to PDR: a rocky road from atomic structures to transport mechanisms. *Int. J. Mol. Sci.* 22:4806. 10.3390/ijms22094806 33946618 PMC8124828

[B25] Kouamo M. F. Ibrahim S. S. Hearn J. Riveron J. M. Kusimo M. Tchouakui M. (2021). Genome-wide transcriptional analysis and functional validation linked a cluster of epsilon Glutathione S-Transferases with insecticide resistance in the major malaria vector *Anopheles funestus* across Africa. *Genes* 12:561. 10.3390/genes12040561 33924421 PMC8069850

[B26] Kretschmer M. Leroch M. Mosbach A. Walker A. S. Fillinger S. Mernke D. (2009). Fungicide-driven evolution and molecular basis of multidrug resistance in field populations of the grey mould fungus *Botrytis cinerea*. *PLoS Pathog.* 5:e1000696. 10.1371/journal.ppat.1000696 20019793 PMC2785876

[B27] Kumar S. Trivedi P. K. (2018). Glutathione S-transferases: role in combating abiotic stresses including arsenic detoxification in plants. *Front. Plant Sci.* 9:751. 10.3389/fpls.2018.00751 29930563 PMC5999759

[B28] Li Q. Wang B. Yu J. Dou D. (2021). Pathogen−informed breeding for crop disease resistance. *J. Integr. Plant Biol.* 63 305–311. 10.1111/jipb.13029 33095498

[B29] Li T. Xiu Q. Zhang J. Wang J. X. Duan Y. B. Zhou M. G. (2020). Pharmacological characteristics and efficacy of fluazinam against *Corynespora cassiicola*, causing cucumber target spot in greenhouses. *Plant Dis.* 104 2449–2454. 10.1094/PDIS-12-19-2649-RE 32579058

[B30] Liang H. J. Di Y. L. Li J. L. Zhu F. X. (2015). Baseline sensitivity and control efficacy of fluazinam against *Sclerotinia sclerotiorum*. *Eur. J. Plant Pathol.* 142 691–699. 10.1007/s10658-015-0644-5

[B31] Liang L. Cheng X. Dai T. Wang Z. Li J. Li X. (2020). Metabolic fingerprinting for identifying the mode of action of the fungicide SYP-14288 on *Rhizoctonia solani*. *Front. Microbiol.* 11:574039. 10.3389/fmicb.2020.574039 33362733 PMC7755717

[B32] Livak K. J. Schmittgen T. D. (2001). Analysis of relative gene expression data using realtime quantitative PCR. *Methods* 25 402–408. 10.1006/meth.2001.1262 11846609

[B33] Lu K. Song Y. Zeng R. (2021). The role of cytochrome P450-mediated detoxification in insect adaptation to xenobiotics. *Curr. Opin. Insect Sci.* 43 103–107. 10.1016/j.cois.2020.11.004 33387688

[B34] Mao X. W. Li J. S. Chen Y. L. Song X. S. Duan Y. B. Wang J. X. (2018). Resistance risk assessment for fluazinam in *Sclerotinia sclerotiorum*. *Pestic. Biochem. Phys.* 144 27–35. 10.1016/j.pestbp.2017.10.010 29463405

[B35] Massi F. Torriani S. F. Borghi L. Toffolatti S. L. (2021). Fungicide resistance evolution and detection in plant pathogens: *Plasmopara viticola* as a case study. *Microorganisms* 9:119. 10.3390/microorganisms9010119 33419171 PMC7825580

[B36] Oakley A. (2011). Glutathione transferases: a structural perspective. *Drug Metab. Rev.* 43 138–151. 10.3109/03602532.2011.558093 21428697

[B37] Omrane S. Sghyer H. Audéon C. Lanen C. Duplaix C. Walker A.-S. (2015). Fungicide efflux and the MgMFS1 transporter contribute to the multidrug resistance phenotype in *Zymoseptoria tritici* field isolates. *Environ. Microbiol.* 17 2805–2823. 10.1111/1462-2920.12781 25627815

[B38] Ons L. Bylemans D. Thevissen K. Cammue B. (2020). Combining biocontrol agents with chemical fungicides for integrated plant fungal disease control. *Microorganisms* 8:1930. 10.3390/microorganisms8121930 33291811 PMC7762048

[B39] Panini M. Tozzi F. Zimmer C. T. Bass C. Field L. Borzatta V. (2017). Biochemical evaluation of interactions between synergistic molecules and phase I enzymes involved in insecticide resistance in B- and Q-type *Bemisia tabaci* (Hemiptera: Aleyrodidae). *Pest Manag. Sci.* 73 1873–1882. 10.1002/ps.4553 28195678

[B40] Pavlidi N. Vontas J. Van Leeuwen T. (2018). The role of glutathione S-transferases (GSTs) in insecticide resistance in crop pests and disease vectors. *Curr. Opin. Insect Sci.* 27 97–102. 10.1016/j.cois.2018.04.007 30025642

[B41] Perlin M. H. Andrews J. San Toh S. (2014). Essential letters in the fungal alphabet: ABC and MFS transporters and their roles in survival and pathogenicity. *Adv. Genet.* 85 201–253. 10.1016/B978-0-12-800271-1.00004-4 24880736

[B42] Perperopoulou F. Pouliou F. Labrou N. E. (2017). Recent advances in protein engineering and biotechnological applications of glutathione transferases. *Crit. Rev. Biotechnol.* 38 511–528. 10.1080/07388551.2017.1375890 28936894

[B43] Pochapsky T. C. Wong N. Zhuang Y. Futcher J. Pandelia M. E. Teitz D. R. (2018). NADH reduction of nitroaromatics as a probe for residual ferric form high-spin in a cytochrome P450. *BBA Proteins Proteom.* 1866 126–133. 10.1016/j.bbapap.2017.04.003 28473297 PMC5665732

[B44] Qu X. P. Li J. S. Wang J. X. Wu L. Y. Wang Y. F. Chen C. J. (2018). Effects of the dinitroaniline fungicide fluazinam on *Fusarium fujikuroi* and rice. *Pestic. Biochem. Phys.* 152 98–105. 10.1016/j.pestbp.2018.09.010 30497718

[B45] Raymaekers K. Ponet L. Holtappels D. Berckmans B. Cammue B. P. (2020). Screening for novel biocontrol agents applicable in plant disease management-a review. *Biol. Control* 144:104240. 10.1016/j.biocontrol.2020.104240

[B46] Rebollar-Alviter A. Madden L. V. Jeffers S. N. Ellis A. A. (2007). Baseline and differential sensitivity to two QoI fungicides among isolates of *Phytophthora cactorum* that cause leather rot and crown rot on strawberry. *Plant Dis.* 91 1625–1637. 10.1094/PDIS-91-12-1625 30780602

[B47] Richter O. Langemann D. Beffa R. (2016). Genetics of metabolic resistance. *Math. Biosci.* 279 71–82. 10.1016/j.mbs.2016.07.005 27424952

[B48] Ristaino B. J. Pamela K. Anderson P. K. Bebber D. P. Kate A. Brauman K. A. (2021). The persistent threat of emerging plant disease pandemics to global food security. *Proc. Natl. Acad. Sci. U.S.A.* 118:e2022239118. 10.1073/pnas.2022239118 34021073 PMC8201941

[B49] Samaras A Ntasiou P. Myresiotis C. Karaoglanidis G. (2020). Multidrug resistance of *Penicillium expansum* to fungicides: whole transcriptome analysis of MDR strains reveals overexpression of efflux transporter genes. *Int. J. Food Microbiol.* 335:108896. 10.1016/j.ijfoodmicro.2020.108896 33070085

[B50] Sang H. Hulvey J. Popko J. T. Lopes J. Swaminathan A. Chang T. (2015). A pleiotropic drug resistance transporter is involved in reduced sensitivity to multiple fungicide classes in *Sclerotinia homoeocarpa* (FT B ennett). *Mol. Plant Pathol.* 16 251–261. 10.1111/mpp.12174 25040464 PMC6638355

[B51] Sang H. Hulvey J. P. Green R. Xu H. Im J. Chang T. (2018). A xenobiotic detoxification pathway through transcriptional regulation in filamentous fungi. *MBio* 9:e00457-18. 10.1128/mBio.00457-18 30018104 PMC6050962

[B52] Schepers H. T. A. M. Kessel G. J. T. Lucca F. Förch M. G. Van Den Bosch G. B. M. Topper C. G. (2018). Reduced efficacy of fluazinam against *Phytophthora infestans* in the Netherlands. *Eur. J. Plant Pathol.* 151 947–960. 10.1007/s10658-018-1430-y 30996524 PMC6435203

[B53] Shao W. Zhang Y. Ren W. Chen C. (2015). Physiological and biochemical characteristics of laboratory induced mutants of *Botrytis cinerea* with resistance to fluazinam. *Pestic. Biochem. Phys.* 117 19–23. 10.1016/j.pestbp.2014.10.003 25619907

[B54] Stergiopoulos I. de Waard M. A. (2002). Activity of azole fungicides and ABC transporter modulators on *Mycosphaerella graminicola*. *J. Phytopathol.* 150 313–320. 10.1046/j.1439-0434.2002.00761.x

[B55] Takahashi J. P. F. Feliciano L. M. Santos D. C. S. Ramos S. Oliveira R. A. Attili-Angelis D. (2020). Could fungicides lead to azole drug resistance in a cross-resistance manner among environmental Cryptococcus strains? *Curr. Fungal Infect. R.* 14 9–14. 10.1007/s12281-020-00373-8

[B56] Tamura O. (2000). “Resistance development of gray mold on beans towards fluazinam and relevant countermeasures,” in *Abstracts of 10^th^ Symposium of Research Committee of Fungicide Resistance*, (Okoyama: Springer), 7–16.

[B57] Tleuova A. B. Wielogorska E. Talluri V. P. Štìpánek F. Elliott C. T. Grigoriev D. O. (2020). Recent advances and remaining barriers to producing novel formulations of fungicides for safe and sustainable agriculture. *J. Control. Release* 326 468–481. 10.1016/j.jconrel.2020.07.035 32721524

[B58] van Bruggen A. H. Gamliel A. Finckh M. R. (2016). Plant disease management in organic farming systems. *Pest Manag. Sci.* 72 30–44. 10.1002/ps.4145 26331771

[B59] Vitoratos A. G. (2014). Mode of action and genetic analysis of resistance to fluazinam in *Ustilago maydis*. *J. Phytopathol.* 162 737–746. 10.1111/jph.12254

[B60] Wadley F. M. (1945). *The Evidence Required to Show Synergistic Action of Insecticides and a Short Cut in Analysis.* Washington DC: USDA, 1–8.

[B61] Wang H. Ping H. Liu Q. Han P. Guo X. (2021). Determination of pesticide residues in strawberries by ultra-performance liquid chromatography-tandem mass spectrometry. *Food Anal. Methods* 15 85–95. 10.1007/s12161-021-02102-4

[B62] Wang Z. Dai T. Peng Q. Gao X. Zhong S. Gao H. (2020). Bioactivity of the novel fungicide SYP-14288 against plant pathogens and the study of its mode of action based on untargeted metabolomics. *Plant Dis.* 104 2086–2094. 10.1094/PDIS-01-20-0142-RE 32544002

[B63] Wang Z. Ni X. Peng Q. Hou Y. Fang Y. Mu W. (2018). The novel fungicide SYP-14288 acts as an uncoupler against *Phytophthora capsici*. *Pestic. Biochem. Phys.* 147 83–89. 10.1016/j.pestbp.2018.01.014 29933997

[B64] Xu C. Li C. Y. T. Kong A. N. T. (2005). Induction of phase I, II and III drug metabolism/transport by xenobiotics. *Arch. Pharm. Res.* 28 249–268. 10.1007/BF02977789 15832810

[B65] Yang X. Deng S. Wei X. Yang J. Zhao Q. Yin C. (2020). MAPK-directed activation of the whitefly transcription factor CREB leads to P450-mediated imidacloprid resistance. *Proc. Natl. Acad. Sci. U.S.A.* 117 10246–10253. 10.1073/pnas.1913603117 32327610 PMC7229646

[B66] Zhang C. Meng D. Wang W. Dai T. Wang J. Guan A. (2020). Overexpression of three P450 genes is responsible for resistance to novel pyrimidine amines in *Magnaporthe oryzae*. *Pest Manag. Sci.* 76 4268–4277. 10.1002/ps.5991 32638503

