# Supplementary material for: Cytochrome P450 and Glutathione S-Transferase Confer Metabolic Resistance to SYP-14288 and Multi-Drug Resistance in Rhizoctonia solani
Source: Front Microbiol. 2022 Mar 21;13:806339. doi: 10.3389/fmicb.2022.806339 (PMC8977892; doi:10.3389/fmicb.2022.806339)
Supplement: Supplementary file 1 [file Table_1.docx]

**Table S1.** Primers used in quantitative real-time polymerase chain reaction (qRT-PCR) for relative quantification of gene expression

| Gene ID | Function annotation | Primers | Sequence (5' to 3') |
| --- | --- | --- | --- |
| AG1IA_05136 | Cytochrome P450 | Forward | CTTGTCAGGGAGAGGTCAAAG |
|  |  | Reverse | CGATAGACTTGGGAGGGAAATG |
| AG1IA_01023 | Cytochrome P450 | Forward | CGCTGTGCTGGTCGAATAA |
|  |  | Reverse | CGCCCTGAGAGTGTAAGAATAG |
| AG1IA_05092 | Cytochrome P450 | Forward | GATGGAGGACAAGGAGATTCAG |
|  |  | Reverse | TAGTGAGAGCAGGCAGAAATG |
| AG1IA_07129 | Cytochrome P450 | Forward | CCTCGTTTCTGTCCTCACATAC |
|  |  | Reverse | GCTCGTAAATGCCCAATCAAAG |
| AG1IA_07929 | Cytochrome P450 | Forward | CGCACCGGATATTGAGAAAGA |
|  |  | Reverse | TACAGTGCCCATGATGTTGTAG |
| AG1IA_06336 | Cytochrome P450 | Forward | GGCACAACAGGAGATTGATTTG |
|  |  | Reverse | CATCGCAGCACTTGGAGTAT |
| AG1IA_07383 | Glutathione S-transferases | Forward | TCCCAAGGCTTATGGGTTATTC |
|  |  | Reverse | CGCATGAAGATTCTCGAGGTAG |
| AG1IA_00711 | Glutathione S-transferases | Forward | CAACCACATTACCCTCGCCT |
|  |  | Reverse | TGGCCTTCCAAGACGAGAAC |
| β-actin | Reference gene | Forward | GCGTGAAGTAGTGCGGGATA |
|  |  | Reverse | AAGTCGGGAGAAAGGAGC |
| GAPDH | Reference gene | Forward | TACTCCGCAATGCTATCG |
|  |  | Reverse | TACTCGGTCCCAGTGGT |
