# Supplementary material for: Cytochrome P450 and Glutathione S-Transferase Confer Metabolic Resistance to SYP-14288 and Multi-Drug Resistance in Rhizoctonia solani
Source: Front Microbiol. 2022 Mar 21;13:806339. doi: 10.3389/fmicb.2022.806339 (PMC8977892; doi:10.3389/fmicb.2022.806339)
Supplement: Supplementary file 2 [file Table_2.docx]

**Table S2**. Quality assessment of reads generated from RNA sequences of wild-type strain X19 and its derived SYP-14288-resistant mutant X19-7 of *Rhizoctonia solani*

| Sample | Raw data read | Raw data base (G) | Valid data read | Valid data base (G) | Valid ratio (%) | Q20 (%) | Q30 (%) | GC content (%) |
| --- | --- | --- | --- | --- | --- | --- | --- | --- |
| X19_1 | 45925986 | 6.89 | 45087248 | 6.76 | 98.17 | 99.98 | 98.51 | 52 |
| X19_2 | 44564978 | 6.68 | 43759966 | 6.56 | 98.19 | 99.98 | 98.51 | 52 |
| X19_3 | 43100056 | 6.47 | 42120750 | 6.32 | 97.73 | 99.97 | 98.41 | 52 |
| X19-7_1 | 49471414 | 7.42 | 48665102 | 7.30 | 98.37 | 99.97 | 98.39 | 52 |
| X19-7_2 | 52854736 | 7.93 | 51953254 | 7.79 | 98.29 | 99.97 | 98.43 | 52 |
| X19-7_3 | 51968498 | 7.80 | 50927812 | 7.64 | 98.00 | 99.98 | 98.46 | 52 |
| X19T_1 | 43510328 | 6.53 | 42737330 | 6.41 | 98.22 | 99.97 | 98.57 | 52 |
| X19T_2 | 47767754 | 7.17 | 46873304 | 7.03 | 98.13 | 99.97 | 98.43 | 52 |
| X19T_3 | 49775208 | 7.47 | 48806130 | 7.32 | 98.05 | 99.97 | 98.53 | 52 |
| X19-7T_1 | 49697340 | 7.45 | 48555732 | 7.28 | 97.70 | 99.98 | 98.55 | 52 |
| X19-7T_2 | 44051694 | 6.61 | 43094274 | 6.46 | 97.83 | 99.98 | 98.50 | 52 |
| X19-7T_3 | 52636178 | 7.90 | 51072854 | 7.66 | 97.03 | 99.98 | 98.40 | 52 |
